# Accelerated maturation of brain circuits and executive function in adolescents who experienced the COVID-19 pandemic

**DOI:** 10.1162/IMAG.a.1307

**Published:** 2026-07-22

**Authors:** Neva M. Corrigan, Elizabeth Huber, Ariel Rokem, T. Christina Zhao, Patricia K. Kuhl

**Affiliations:** Institute for Learning & Brain Sciences, University of Washington, Seattle, WA, United States; Institute on Human Development and Disability, University of Washington, Seattle, WA, United States; Department of Psychology, University of Washington, Seattle, WA, United States; eScience Institute, University of Washington, Seattle, WA, United States; Department of Speech and Hearing Sciences, University of Washington, Seattle, WA, United States

**Keywords:** COVID-19 pandemic, executive function, white matter development, adolescent brain, diffusion-weighted MRI

## Abstract

The COVID-19 pandemic disrupted many aspects of daily life. Previous studies have suggested that these disruptions altered the trajectory of typical adolescent gray matter development. This study examined whether development of white matter and of executive function (EF), measured behaviorally, were similarly affected. Diffusion-weighted MRI (dMRI) and EF measurements were collected at two timepoints: pre-pandemic and post-lockdown. Mean diffusivity and fractional anisotropy were calculated from the dMRI data. Normative models calculated from a pre-pandemic adolescent sample were used to assess deviations from typical development in a separate post-lockdown sample from the same participant cohort. Several white matter pathways showed accelerated development post-lockdown, and the acceleration was greater in females than in males. Post-lockdown performance on EF tasks also showed accelerated development. These findings suggest that the COVID-19 pandemic was associated with both accelerated white matter maturation and accelerated EF performance.

## 1 Introduction

Adolescence is a period in which the brain undergoes substantial changes in structure to support development in social, emotional, and cognitive functioning (Konrad et al., 2013). Much of this development is shaped by the external environment. The COVID-19 pandemic prompted governments around the world to enact confinement and social distancing policies as public health interventions aimed at reducing viral transmission and limiting severe health outcomes and mortality. These measures resulted in dramatic disruptions to the daily routines of millions of individuals, which had far-reaching effects on many aspects of life including academic learning environments, extracurricular activities, and interpersonal interactions. Numerous studies have reported detrimental effects of the COVID-19 pandemic on adolescent mental health (for example, Kiss et al., 2022; Thorisdottir et al., 2023), and others have reported alterations in adolescent brain structural development. Accelerated maturation of cerebral gray matter in adolescents associated with the COVID-19 pandemic has been reported by our group and by others (Corrigan et al., 2024; Gotlib et al., 2023; van Drunen et al., 2023). This accelerated maturation was evidenced by greater cortical thinning in post-COVID adolescents than expected for their age. Alterations to the development of the hippocampus and the amygdala have also been reported (Gotlib et al., 2023; van Drunen et al., 2023). In the present study, we investigated how the pandemic affected white matter development.

White matter makes up almost half of the volume of the brain (Paus, 2010). Whereas gray matter is comprised of neuronal cell bodies, dendrites, and short-distance cortical white matter connections, white matter tracts are comprised of myelinated axons that originate from the neuronal cell bodies and traverse long distances to allow for neural impulses to be transmitted between regions of the brain that are in different spatial locations and have different specialized functions. Maturation of these white matter pathways across childhood, adolescence, and early adulthood is essential for maturation of cognitive, motor, and sensory skills (Paus, 2010). To our knowledge, only one study has evaluated the effect of the COVID-19 pandemic on white matter development. Blockmans et al. (2025) investigated the maturation of tracts associated with reading (the bilateral arcuate fasciculi and the bilateral inferior fronto-occipital fasciculi) at two timepoints: the end of kindergarten (before COVID-19 pandemic-related school closures) and the end of second grade (after schools reopened). The authors reported no effect of school closures on the maturation of these white matter tracts.

Diffusion-weighted magnetic resonance imaging (dMRI) provides detailed information about the development of white matter in the brain. The dMRI measurement is sensitive to the magnitude and direction of water diffusion, and since characteristics of white matter fibers, including their orientation, density, and degree of myelination, affect the diffusion of water, dMRI is used to infer both the trajectory of major white matter bundles, as well as the microstructural properties of the tissue within them. Based on the diffusion tensor model (Basser et al., 1994; Basser & Pierpaoli, 1996), two key metrics can be calculated to evaluate white matter microstructural characteristics: mean diffusivity (MD) and fractional anisotropy (FA). MD provides a measure of the magnitude, or average amount, of water diffusion occurring in all directions. FA reflects the degree to which this diffusion is restricted in direction (Basser & Pierpaoli, 1996; Kochunov et al., 2011; Thomason & Thompson, 2011). Changes in these metrics across childhood and adolescent development have been well characterized, where MD has been found to decrease across development and FA has been found to increase (Lebel et al., 2012; Lebel & Beaulieu, 2011; Luo et al., 2026; Tamnes et al., 2010). These changes are thought to reflect increases in axonal myelination, the number of axons, and in axonal caliber across development.

Numerous studies have reported sex differences in the mental health impact of the pandemic (e.g., Halldorsdottir et al., 2021; Kiss et al., 2022; Turner et al., 2023). For example, a review of longitudinal studies of children and adolescents reported that after the pandemic, females were at a higher risk of experiencing internalizing symptoms, anxiety and depression, feelings of stress, and feelings of poor well-being as compared to pre-pandemic levels (Wolf & Schmitz, 2024). Males were more at risk for attention problems, addictive gameplay, and decreases in feeling of life satisfaction (Wolf & Schmitz, 2024). We have reported significant sex differences in the impact of the COVID-19 pandemic on the development of cortical gray matter (Corrigan et al., 2024).

Adolescence is an important period in the development of executive function (EF), a domain of cognitive functioning that involves the ability to control one’s thoughts, feelings, and actions (Crone, 2009). EF plays an essential role in shaping an individual’s social, psychological, and cognitive development, mental and physical health, academic achievement, quality of life, and long-term life outcomes (Diamond, 2013; Nyongesa et al., 2019). EF is comprised of a set of skills that include selective attention, decision-making, response inhibition, and working memory (Blakemore & Choudhury, 2006). These skills are immature during childhood and develop throughout adolescence and early adulthood. It is thought that improvements in these skills during adolescence are related to structural changes in the frontal lobe (Blakemore & Choudhury, 2006), including in white matter regions (Bagautdinova et al., 2023; Goddings et al., 2021).

Studies examining the impact of the COVID-19 pandemic on EFs in children and adolescents have largely reported adverse effects. Attwood and Jarrold (2023) reported a decrease in self-perceived attention, planning, and task completion abilities in adolescents aged 16–18 years during the lockdown period. Similarly, Tabullo et al. (2023) found that parents of children aged 9–11 years reported decreased cognitive flexibility associated with the pandemic lockdowns. A study of the effects of confinement on parent reports of EF in children and adolescents aged 6–18 years early in the confinement period of the pandemic reported significant declines in EF (Lavigne-Cerván et al., 2021). A follow-up study of the same sample reported improvements after the end of the confinement period (Navarro-Soria et al., 2023). A longitudinal study of data collected in-person both pre- and post-pandemic in adolescents aged 12 years reported impairment in one test of EF in males, but no effects in females (Rivera-Urbina et al., 2025). Reported negative effects on EF may not reflect persistent impairment. Some of these effects may have been transient in nature and may not have accounted for recovery or developmental catch-up beyond the time span of the study. In addition, longitudinal studies of executive function are subject to practice effects as a potential confounding factor.

The present study was originally designed to evaluate longitudinal change in typical development by assessing within-subject change over time. However, the COVID-19 pandemic occurred between the two data collection timepoints, meaning all study participants were unexpectedly exposed to a common environmental disruption between measurements. As a result, within-subject changes could not be interpreted as reflecting typical development alone, as they were potentially confounded by pandemic-related effects. Since there was no control (non-pandemic-exposed) group in our study sample, traditional modeling of within-subject longitudinal change could not disentangle processes related to typical development from pandemic-related influences. For this reason, we adopted a strategy based on normative modeling that allowed us to explore deviations from normative adolescent development after exposure to the pandemic environment. Normative modeling is a statistical technique that can reveal whether measurements deviate from typical age-related development (Marquand et al., 2016, 2019). In this technique, a “normative model” of the expected trajectory of change with age is created from a sample of typically developing individuals. Data for individuals from a different sample are compared to the normative model to evaluate the degree of any deviation from typical development. This approach allowed us to use all of our study data, but could not make use of the longitudinal nature of the data. Instead, the analysis was cross-sectional. Normative modeling has been utilized to examine the effects of socioeconomic disadvantage (Rakesh et al., 2021), traumatic stress (Wong et al., 2023), as well as developmental disorders (Jiang et al., 2024; Shan et al., 2022; Wolfers et al., 2019; Zabihi et al., 2019, 2020) on brain structure during development and across the lifespan. Normative models (also known as normative data or normative standards) are also commonly used to evaluate individual performance relative to a typical population on neurocognitive batteries such as the Mullen Scales of Early Learning (Mullen, 1995) and the Wechsler Intelligence Scale for Children (Wechsler, 2014). In a previous study, we used normative modeling to assess the effects of the COVID-19 pandemic on the development of cerebral gray matter in an adolescent sample (Corrigan et al., 2024).

The purpose of the current study is to investigate whether the COVID-19 pandemic was associated with alterations in white matter development in an adolescent sample, whether they affected development of EF in the same sample, and to investigate the pattern of association between these two measures. We hypothesized that in the post-lockdown data we would see 1) altered development in white matter tracts throughout the brain, 2) differing effects on white matter according to sex, 3) altered maturation of executive function abilities, and 4) relationships between the degree of altered white matter development and deviations in EF measures from typical developmental trajectories.

## 2 Materials and Methods

Data were acquired from adolescents longitudinally at two timepoints: in 2018, prior to the pandemic, and then 3 years later, starting in August of 2021 and continuing into early 2022 (see Supplemental Materials Table S1 for how these dates compare to the COVID-19 pandemic restrictive measure dates in Washington State). Details of this sample are also described in a previous study from our lab (Corrigan et al., 2024). All study subjects were recruited from the local community. The ages of the participants at the pre-pandemic timepoint were 9, 11, 13, 15, and 17 years. The ages of participants at the post-pandemic timepoint were 12, 14, 16, 18 and 20 years. Participants were excluded from the study if they were left-hand dominant (as determined by the Edinburgh Handedness Inventory); if English was not the primary language spoken in the home; if they had any history of speech, language, or hearing difficulties; if they had an uncorrected vision problem; if they had ever been diagnosed with a developmental or psychiatric disorder; if they had any surgical implants or dental work that could interfere with the MRI; if they identified as a different gender than that assigned at birth; or if they were taking psychotropic medications.

At the first timepoint (pre-COVID-19 pandemic), behavioral data were collected from a total of 164 subjects (81F; 33 nine-year-olds, 33 eleven-year-olds, 35 thirteen-year-olds, 31 fifteen-year-olds, 32 seventeen-year-olds). MR data were collected from 163 subjects (81F; 32 nine-year-olds, 33 eleven-year-olds, 35 thirteen-year-olds, 31 fifteen-year-olds, and 32 seventeen-year-olds). Data from 1 seventeen-year-old were excluded due to an incidental finding. At the second timepoint (post-lockdown), behavioral data were collected from a total of 137 subjects (70F; 28 twelve-year-olds, 28 fourteen-year-olds, 32 sixteen-year-olds, 23 eighteen-year-olds, and 26 twenty-year olds). MRI data were collected from 131 subjects (65F; 27 twelve-year-olds, 25 fourteen-year-olds, 31 sixteen-year-olds, 23 eighteen-year-olds, and 25 twenty-year-olds). Socioeconomic status (SES) was assessed using the Hollingshead Index (Hollingshead, 2011). All study procedures were approved by the UW Human Subjects Board, and informed consent was obtained from each participant and a parent.

### 2.1 Behavioral data acquisition

The NIH Toolbox Cognition Battery (v.2.1) was administered to all study participants at both timepoints. Standard scores uncorrected for age were utilized in all analyses. Scores are scaled to a mean of 100 and standard deviation of 15 (National Institutes of Health & Northwestern University, 2016). Full details of the normative sample are described in Weintraub et al. (2013). Its measures of executive function include the dimensional change card sort test (DCCS), flanker inhibitory control and attention test, and the list sorting working memory test. At timepoint 1 (prior to the COVID-19 pandemic), all assessments were conducted in person on an iPad Air device. At timepoint 2, only the DCCS and the flanker test were administered in person. The working memory test data were excluded from the analysis because the test was not administered in person at both timepoints. The demographics of the subjects whose flanker and DCCS data were included in the analysis are shown in Tables 1 and 2.

**Table 1.** Participant demographics and executive function scores at the pre-COVID-19 timepoint.

	sample size		Mean age ± SD		Mean SES ± SD		Mean flanker ± SD		Mean DCCS ± SD	
Age group (years)	Male	Female	Male	Female	Male	Female	Male	Female	Male	Female
9	17	16	9.1 ± 0.03	9.1 ± 0.03	55.9 ± 5.5	52.9 ± 9.7	97.1 ± 6.2	92.1 ± 9.2	94.1 ± 14.8	88.8 ± 8.0
11	17	16	11.1 ± 0.05	11.1 ± 0.05	55.6 ± 9.3	55.3 ± 6.8	99.1 ± 9.1	100.9 ± 9.1	99.5 ± 8.9	101.0 ± 8.5
13	18	17	13.1 ± 0.05	13.1 ± 0.04	52.1 ± 9.8	54.8 ± 9.5	104.1 ± 5.4	101.7 ± 6.8	109.9 ± 6.6	108.1 ± 8.0
15	15	16	15.1 ± 0.03	15.1 ± 0.05	53.6 ± 8.3	54.1 ± 10.4	109.9 ± 4.9	105.7 ± 7.2	114.7 ± 4.8	112.1 ± 6.4
17	16	16	17.1 ± 0.05	17.1 ± 0.06	54.6 ± 7.7	53.9 ± 10.2	109.3 ± 7.4	105.7 ± 5.9	113.2 ± 6.2	109.6 ± 13.3
Total	83	81								

Flanker: Flanker standard score uncorrected for age; DCCS: Dimensional Change Card Sort standard score uncorrected for age. Data are shown for all subjects included in the behavioral data analysis.

**Table 2.** Participant demographics and executive function scores at the post-lockdown timepoint.

	Sample size		Mean age ± SD		Mean SES ± SD		Mean flanker ± SD		Mean DCCS ± SD	
Age group (years)	Male	Female	Male	Female	Male	Female	Male	Female	Male	Female
12	15	13	12.3 ± 0.18	12.2 ± 0.18	56.1 ± 5.7	52.5 ± 10.1	105.8 ± 6.5	105.6 ± 5.4	106.2 ± 15.4	107.3 ± 7.0
14	14	14	14.3 ± 0.24	14.4 ± 0.17	55.2 ± 10.2	57.0 ± 4.8	108.5 ± 9.3	111.6 ± 4.6	111.1 ± 8.7	111.4 ± 5.6
16	16	16	16.4 ± 0.20	16.4 ± 0.27	52.9 ± 10.1	55.3 ± 9.5	113.5 ± 4.1	109.8 ± 8.0	115.5 ± 4.3	114.1 ± 6.0
Total	45	43								

Flanker: Flanker standard score uncorrected for age; DCCS: Dimensional Change Card Sort standard score uncorrected for age. Data are shown for all subjects included in the behavioral data analysis.

The Eriksen flanker task in the NIH Toolbox assesses selective attention and inhibitory control (Eriksen & Eriksen, 1974; Weintraub et al., 2013). In this task, there is a “target” arrow pointing to the left or right in the middle of the screen. This arrow is flanked by “non-target” arrows on the left- and right-hand side of the target arrow. The objective of the task is to press a left or right arrow key that corresponds to the target arrow’s direction. There are three types of non-target stimuli in this task: congruent, incongruent, and neutral. When the non-target stimuli are congruent, the direction of the non-target stimuli arrows is the same as the target arrow. In the incongruent task, the non-target arrows point in the opposite direction to that of the target arrow. In the neutral mode, the flanking stimuli are not arrows; they do not point in the same or opposite direction from the target stimulus. The reaction time and accuracy of the responses are recorded for scoring. Higher scores on this task signify better performance (Baghdadi et al., 2021).

The DCCS task (Zelazo, 2006) assesses cognitive flexibility as well as elements of attention, working memory, and inhibition (Doebel & Zelazo, 2015). In this test, an image is shown on the top half of the screen, and two images are shown on the bottom half of the screen. The objective of the task is to touch the image at the bottom of the screen that matches the image at the top of the screen in a specified dimension (either shape or color). In the initial stage of the experiment, one dimension is specified. In the post-switch stage, the dimension to select on is switched (i.e., from color to shape or vice versa; Doebel & Zelazo, 2015). Higher scores on this task signify better performance.

### 2.2 MRI data acquisition

MR data were acquired on a 3.0 T Philips Ingenia MRI system. There were no hardware or software changes during the study period, including consistent use of the same 32-channel phased-array receiver coil, and software version (R541). A Pearltec Crania (Pearltec AG, Schlieren/Zurich, Switzerland) head fixation system was used to minimize head motion. High resolution T1-weighted images of the head were acquired using a multi-echo MPRAGE sequence with FOV = 240 × 240 × 200, acquisition voxel size 1.0 × 1.0 × 1.0 mm, reconstructed voxel size 0.5 × 0.5 × 0.5 mm, TR/TI/TE1/TE2 = 13.6/1100/3.7/9.8 ms, shot interval 2200 ms, and flip angle (FA) = 12°. dMRI data were acquired using a single-shot DWI-EPI sequence with FOV = 240 × 240 × 140 mm, voxel size 1.5 × 1.5 × 2 mm, TR/TE = 8600/88.77 ms. Each diffusion scan included 6 non-diffusion-weighted (b = 0) volumes and 64 diffusion-weighted volumes acquired with a b-value of 2000 s/mm^2^ (50 non-collinear gradient directions) or 800 s/mm^2^ (12 non-collinear gradient directions). An additional set of 6 non-diffusion-weighted volumes was acquired using the same parameters but a reversed-phase encoding direction (posterior-anterior), for use in correcting EPI distortions (Andersson et al., 2003).

### 2.3 Quality control

Before pre-processing, quality assurance of both the raw diffusion-weighted and the b = 0 images was performed using a 3-point scale, with 0 indicating severe artifacts affecting multiple volumes; 1 indicating acceptable image quality with minor artifacts; and 2 indicating excellent image quality. Datasets containing any image with a quality grade of 0 were excluded from processing. In addition, at the first timepoint, diffusion data acquisition for one participant was not completed due to a receiver coil error, and one participant was excluded due to a metal artifact. At the second timepoint, one participant was unable to complete the diffusion scans. Tables 3 and 4 show the demographics of the subjects included in the dMRI analysis.

**Table 3.** Participant demographics for the brain imaging analysis at the pre-COVID-19 timepoint.

	Sample size		Mean age ± SD		Mean SES ± SD	
Age group (years)	Male	Female	Male	Female	Male	Female
9	11	13	9.1 ± 0.03	9.1 ± 0.04	55.5 ± 5.2	52.4 ± 10.8
11	13	14	11.1 ± 0.05	11.1 ± 0.05	55.6 ± 8.8	56.4 ± 5.2
13	8	10	13.1 ± 0.06	13.1 ± 0.04	50.5 ± 11.0	53.6 ± 10.5
15	9	13	15.2 ± 0.02	15.1 ± 0.05	51.4 ± 9.3	54.4 ± 11.4
17	14	15	17.1 ± 0.04	17.1 ± 0.06	54.1 ± 8.1	53.2 ± 10.2
Total	55	65				

**Table 4.** Participant demographics for the brain imaging analysis at the post-lockdown timepoint.

	Sample size		Mean age ± SD		Mean SES ± SD	
Age group (years)	Male	Female	Male	Female	Male	Female
12	14	11	12.3 ± 0.19	12.2 ± 0.14	55.5 ± 5.4	52.5 ± 11.0
14	11	11	14.4 ± 0.25	14.4 ± 0.18	55.0 ± 11.0	56.4 ± 4.6
16	11	11	16.4 ± 0.20	16.4 ± 0.27	52.4 ± 10.1	52.0 ± 9.1
Total	36	33				

### 2.4 dMRI data analysis

The dMRI data were preprocessed using the FSL BET tool for brain extraction (Smith, 2002), FSL TOPUP for correction of susceptibility-induced distortions (Andersson et al., 2003; Smith et al., 2004), and FSL EDDY (Andersson & Sotiropoulos, 2016) for correction of head motion and eddy current-induced distortions. For FSL TOPUP, a single pair of b = 0 images with opposite phase-encoding directions was used to estimate the susceptibility-induced off-resonance field. Diffusion-weighted volumes were aligned to an average of the non-diffusion-weighted volumes in each scan using rigid body registration (SPM v.12; Ashburner & Friston, 1997) and resampled to 2 mm isotropic resolution using image processing tools within the VISTASOFT package (http://github.com/vistalab/vistasoft). Diffusion gradients were adjusted to account for transformations applied during image registration and motion (Leemans & Jones, 2009). The mrDiffusion tools within the VISTASOFT package were used to perform tensor fitting, to generate whole-brain maps of FA and MD, and to perform deterministic tractography (Basser et al., 2000). The Matlab Automated Fiber Quantification software package (AFQ; Yeatman et al., 2012) was then used to identify and segment 16 major white matter fiber tracts from the JHU white matter atlas (Mori et al., 2005), which includes a total of 20 tracts. The 16 segmented tracts were the left and right thalamic radiations, arcuate fasciculi, inferior fronto-occipital fasciculi (IFOF), superior longitudinal fasciculi (SLF), inferior longitudinal fasciculi (ILF), uncinate fasciculi, corticospinal tracts, and the callosum forceps major and callosum forceps minor. Four tracts in the JHU atlas (the bilateral cingulum cingulate and the bilateral cingulum hippocampus) were not reliably reconstructed in all subjects and thus were not included in the present analysis. Each tract was summarized by a vector of 100 FA or MD values sampled at equidistant locations (nodes) along the tract.

### 2.5 Normative modeling

For both the executive function as well as the dMRI measures, pre-COVID (timepoint 1) data were used to construct normative models representing trajectories of expected change with age during typical adolescent development. The post-lockdown (timepoint 2) data were then compared to these models to assess deviations from what would be expected during typical development. Normative models were constructed using Bayesian linear regression (BLR), with age (in days) and sex included as covariates, and were implemented in Python (v. 3.8) using functions from the Predictive Clinical Neuroscience (PCN) Toolkit (v. 0.27; Rutherford et al., 2022). BLR was chosen for its relative simplicity and scalability (Fraza et al., 2021). The PCN toolkit BLR implementation uses a spline basis. To determine the appropriate model order for both the imaging and the EF data, we compared first-order (linear) and second-order (nonlinear) spline models. Model performance was assessed using five-fold cross-validation and calculation of BIC and RMSE. For the imaging data, after model order was determined, model validity was assessed using a nested validation procedure, performed within each of the 100 train/test splits described below. For each split, the pre-COVID training data were further divided into a training subset and a held-out validation subset, and an interim model was fit on the training subset. The mean and standard deviation of Z-scores, explained variance, root mean square error, and Pearson’s correlation coefficient were calculated for the held-out validation subset. Tracts whose models had average explained variance below 0 across all nodes and splits were considered invalid and excluded from subsequent analyses. The training subset and held-out validation subset were used only for this validation step and were not used in subsequent model fitting.

Normative models were trained on repeatedly resampled subsets of the data (100 iterations) to reduce dependence on any single train-test split. Subjects were split into a “train” set, whose pre-COVID data were used for building the normative model, and a “test” set, whose post-lockdown data were used for assessment of deviation from typical development. To prevent data leakage, pre- and post-lockdown data from the same subject were not used for both normative model construction and post-lockdown evaluation within the same split. Subjects with only timepoint 1 data were always included in the train set, and those with only timepoint 2 data were always included in the test set. Subjects with data at both timepoints were split between train and test sets using a shuffle-split procedure (stratified by age). After each split, subjects in the 18- and 20-year-old age group were removed from the test set because these ages exceeded the range represented in the pre-COVID training data.

For each split, the “estimate” module of the PCN toolkit, with BLR specified as the algorithm and “powell” as the optimization method, was used to generate Z-scores for the test subjects reflecting deviation from the normative model. To ensure that the results are robust to idiosyncratic selection of “train” and “test” participants, we report the mean post-lockdown Z-score for each subject across all test splits they appeared in.

This modeling approach was applied separately to the flanker and DCCS measures. The same approach was then applied separately to the FA and MD values. For the executive function data, there was only one measurement per subject at each timepoint. For the brain imaging data, where there were FA and MD values for each of 100 equidistant nodes for each tract, the 20 nodes at each end of each tract were removed, to reduce partial volume effects with cortex at the ends of the tracts. This resulted in 60 nodes per tract. For both FA and MD, normative modeling was applied separately to each node along each of the 16 tracts, to yield average Z-scores for each node and tract for every subject.

### 2.6 Statistical analysis

For the two EF behavioral measures, the “ttest_1samp” function of the statsmodels.stats.multitest Python library (Seabold & Perktold, 2010) was used to perform a two-tailed one sample *t*-test on the average Z-scores at timepoint 2 for all subjects to calculate whether the mean of the values for all subjects was significantly different from zero (which would indicate that they significantly deviate from the normative model). False discovery rate correction was performed, using the “statsmodels.multipletests” function to correct for multiple comparisons and a significance threshold of 0.05 was utilized. This analysis was performed for all data combined, as well as separately for males and females. The “ttest_ind” function of the statsmodels.stats.multitest library was used to perform an independent two-sample *t*-test to evaluate whether the Z-scores at timepoint 2 differed between males and females.

For the imaging data, findings were based on statistical significance at the tract-level. For within-sex analyses, and for the sex comparison analysis, multiple comparisons correction was applied across tracts using FDR (separately by sex and for FA and MD). Node-wise analyses were conducted for post-hoc descriptive purposes only and were not corrected for multiple comparisons.

A within-sex analysis was performed first to evaluate whether the average Z-score at the post-lockdown timepoint for each sex and tract were significantly different from zero. For this within-sex analyses, a generalized additive model (GAM) was fit to each tract, for FA and MD separately. Statistical inference was performed at the tract level, yielding one *p*-value per tract for each of FA and MD, indicating significance of deviation from the normative model. The GAM models were created using the “tractable_single_tract” function of the Tractable software package v.0.3.0 (Kruper et al., 2025). The models included a smooth function of position along each tract to capture spatial variation, since the values at each node were not independent from each other. Effect sizes were calculated as the mean model-predicted standardized value across tract nodes. For each sex and each metric (FA and MD), false discovery rate (FDR) correction was applied to the tract-level *p*-values to account for multiple comparisons across the 16 tracts. A significance threshold of 0.05 was used. An additional analysis was performed using the same methods, but with male and female data combined.

For the sex-comparison analysis, GAMs were fit to each tract separately for FA and MD, using the “tractable_single_tract” function of the Tractable software package. This model included sex as a predictor. Sex differences were quantified using the regression coefficient for sex in the models. As the response variables were Z-scores, the resulting coefficients represent mean differences between males and females expressed in standard deviation units and are reported as effect sizes. For each sex and metric, statistical inference was performed at the tract level, and FDR correction was applied to the resulting tract-level *p*-values to account for multiple comparisons across the 16 tracts. A significance threshold of 0.05 was used.

Node-wise *p*-values were calculated as a post-hoc evaluation of spatial effects along tracts that showed significant post-lockdown effects at the tract level. Deviations from normative values at individual nodes were evaluated by inspecting the confidence intervals generated by the “plot_smooth” function of the itsadug software package (van Rij et al., 2020). The sim.ci = TRUE option was used to account for correlation among neighboring nodes of each tract, providing joint confidence bands that effectively reduce the risk of false positives due to multiple comparisons. For descriptive purposes, mean Z-scores were calculated at each node and plotted along tracts showing significant tract-level effects using the “plot_tract_profiles” function of the Tractable software package, with nodes showing significant deviations highlighted based on the node-wise *p*-values. Node-wise *p*-values were used solely to illustrate the spatial distribution of tract-level effects in figures, and no additional multiple comparison correction was applied at the node level.

In an exploratory analysis, we used the “pearsonr” function of the statsmodels toolbox in Python (v. 3.8) to calculate Pearson correlation coefficients between average Z-scores for each subject at timepoint 2 for the flanker and DCCS tasks, and average Z-score at timepoint 2 for FA and MD for all white matter tracts showing significant deviations in the post-lockdown data. FDR correction was utilized to account for multiple comparisons.

## 3 Results

### 3.1 White matter tract development

Model comparison indicated that linear spline models outperformed second-order models across regions and nodes for both FA and MD, as reflected by consistently lower RMSE and BIC values. Across 100 train/validation splits, Z-scores in the held-out pre-COVID validation sets closely approximated a standard normal distribution for both MD (mean = -0.03, SD = 0.98) and FA (mean = 0.03, SD = 0.99). Two tracts were excluded from the MD analysis, and nine tracts were excluded from the FA analysis due to poor model fit (explained variance < 0). Predictive performance metrics for tracts included in the analyses are shown in Supplementary Table S2.

#### 3.1.1 Mean diffusivity

**Fig. 1.**
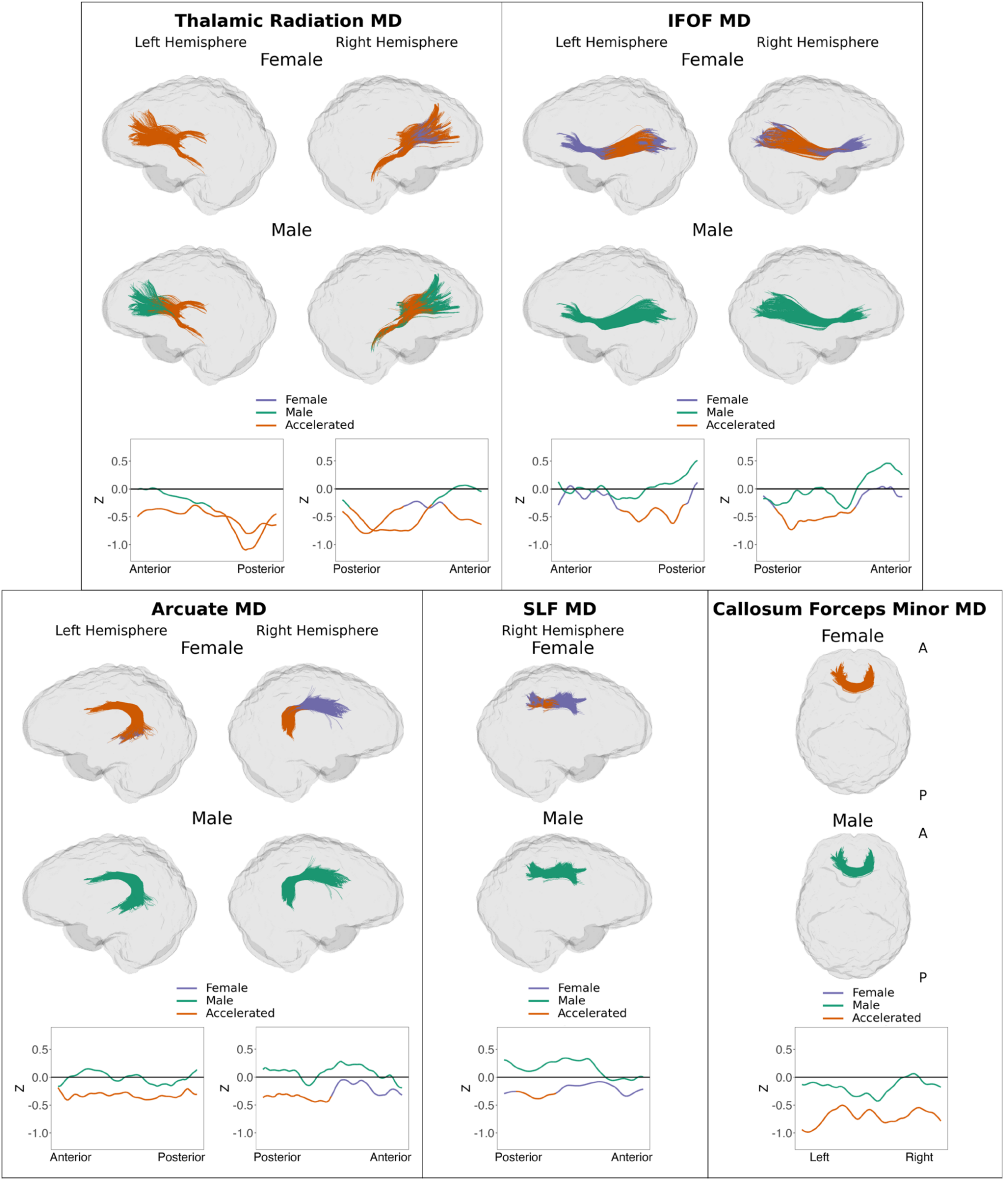
Visualization of post-lockdown mean diffusivity (MD) findings for white matter tracts exhibiting significant pandemic-related effects. Each panel displays the tract streamlines for females (purple) and males (green) overlaid on a 3-D rendering of the brain. The Z-score tract profile, where the x-axis is the node number and the y-axis is the Z-score, is shown at the bottom of each panel. Both male and female brains are depicted for each tract, regardless of whether significant findings were present in both sexes. Nodes exhibiting statistically significant post-lockdown differences (*p* < 0.05) (indicating accelerated development) are highlighted in orange on the tract streamlines and on the tract profile plots.

Findings for MD are shown in Table 5. In females, post-lockdown MD values were found to be significantly lower than predicted by the normative models in the left and right thalamic radiations, the left and right hemisphere inferior fronto-occipital fasciculi (IFOF), the left and right arcuate fasciculi, the right superior longitudinal fasciculus (SLF), and the callosum forceps minor. In males, post-lockdown MD values were found to be significantly lower than predicted by the normative models in the left and right hemisphere thalamic radiations. The spatial locations of the regions along these tracts with significantly lower MD as compared to the pre-COVID data are shown in Figure 1. The MD findings for the analysis with male and female data combined are shown in Supplementary Table S3.

**Table 5.** Mean diffusivity (MD) findings in the post-lockdown data.

	Male			Female			Sex difference		
Tract	Effect size	*p*	FDR	Effect size	*p*	FDR	Effect size	*p*	FDR
Left thalamic radiation	-0.35	0.00	**0.02**	-0.51	0.00	**0.00**	0.16	0.31	0.54
Right thalamic radiation	-0.39	0.00	**0.02**	-0.46	0.00	**0.00**	0.08	0.65	0.73
Left corticospinal	-0.11	0.32	0.64	-0.13	0.14	0.17	0.02	0.91	0.91
Right corticospinal	-0.16	0.18	0.56	-0.22	0.05	0.07	0.08	0.60	0.73
Callosum forceps minor	-0.16	0.25	0.56	-0.71	0.00	**0.00**	0.54	0.01	0.07
Left IFOF	0.01	0.90	0.97	-0.26	0.03	**0.04**	0.25	0.11	0.29
Right IFOF	0.01	0.93	0.97	-0.33	0.00	**0.01**	0.34	0.04	0.16
Left ILF	-0.06	0.68	0.90	-0.22	0.09	0.10	0.14	0.45	0.60
Left SLF	0.01	0.97	0.97	-0.13	0.29	0.29	0.14	0.41	0.60
Right SLF	0.17	0.16	0.56	-0.25	0.01	**0.03**	0.45	0.00	0.06
Left uncinate	-0.16	0.23	0.56	-0.31	0.06	0.09	0.19	0.38	0.60
Right uncinate	-0.06	0.60	0.90	-0.12	0.28	0.29	0.07	0.68	0.73
Left arcuate	-0.01	0.90	0.97	-0.30	0.02	**0.03**	0.29	0.09	0.29
Right arcuate	0.05	0.67	0.90	-0.32	0.01	**0.01**	0.35	0.03	0.14

Bold values indicate statistically significant results after false discovery rate (FDR) correction (adjusted *p* < 0.05).

#### 3.1.2 Fractional anisotropy

**Fig. 2.**
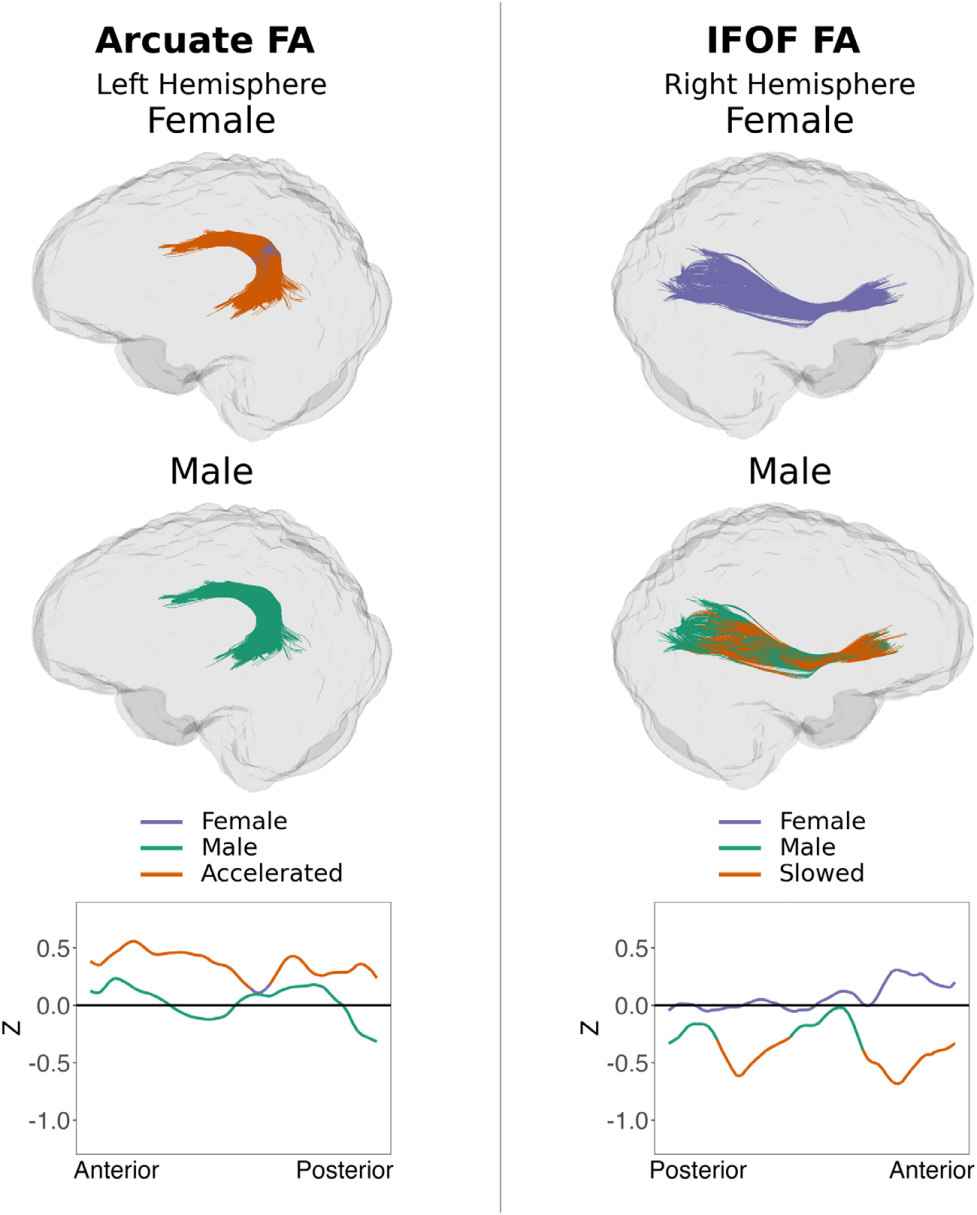
Visualization of post-lockdown fractional anisotropy (FA) findings for white matter tracts exhibiting significant pandemic-related effects. Each panel displays the tract streamlines for females (purple) and males (green), overlaid on a 3-D rendering of the brain. The Z-score tract profile, where the x-axis is the node number and the y-axis is the Z-score, is shown at the bottom of each panel. Both male and female brains are depicted for each tract, regardless of whether significant findings were present in both sexes. Nodes exhibiting statistically significant post-lockdown differences (*p* < 0.05) (indicating either slowed or accelerated development) are highlighted in orange on the tract streamlines and on the tract profile plots.

Findings for FA are shown in Table 6. Post-lockdown FA values were found to be significantly greater than predicted by the normative models in females in the left arcuate fasciculus. In males, post-lockdown FA values were found to be significantly lower than predicted by the normative models in the right hemisphere inferior fronto-occipital fasciculus (IFOF). The spatial locations of these findings are shown in Figure 2. There were no significant FA findings for the analysis of combined male and female data.

**Table 6.** Fractional anisotropy (FA) findings in the post-lockdown data.

	Male			Female			Sex difference		
Tract	Effect size	*p*	FDR	Effect size	*p*	FDR	Effect size	*p*	FDR
Left thalamic Radiation	-0.00	0.94	0.96	0.06	0.58	0.84	-0.05	0.76	0.90
Right corticospinal	-0.01	0.86	0.96	0.01	0.95	0.95	-0.03	0.85	0.90
Callosum forceps Minor	-0.28	0.01	0.07	0.17	0.15	0.48	-0.44	0.01	**0.03**
Right IFOF	-0.34	0.00	**0.00**	0.07	0.46	0.81	-0.41	0.00	**0.02**
Left SLF	-0.05	0.61	0.96	-0.02	0.87	0.95	-0.04	0.78	0.90
Right SLF	-0.00	0.96	0.96	0.15	0.13	0.48	-0.12	0.36	0.58
Left arcuate	-0.02	0.84	0.96	0.34	0.00	**0.03**	-0.35	0.01	0.06

Bold values indicate stastistically significant results after false discovery rate (FDR) correction (adjusted *p* < 0.05).

#### 3.1.3 Sex differences

Results for the evaluation of sex differences are shown in Tables 5 and 6. In the post-lockdown data, deviations from the normative model were found to be higher in females than in males for FA in the callosum forceps minor, and the right hemisphere inferior fronto-occipital fasciculus (IFOF). Z-score distributions of FA for males and females averaged across all 60 nodes for these two tracts are shown in Supplementary Figure S1.

### 3.2 Executive function development

Means and standard deviations for the flanker and DCCS scores used in the analysis at timepoint 1 and timepoint 2 are shown in Tables 1 and 2, respectively. RMSE and BIC indicated that a linear model provided better predictive performance for the flanker task, with lower values for both RMSE and BIC than the nonlinear model. For the DCCS task, RMSE was lower for the linear model whereas BIC was lower for the nonlinear model (∆BIC = 4.3), indicating some ambiguity in optimal model order. Given the consistent advantage of the linear model in RMSE across both tasks, a linear spline model (order 1 with 2 knots) was used in this analysis. A second-order model was evaluated as a sensitivity analysis (reported in the Supplementary Materials).

**Fig. 3.**
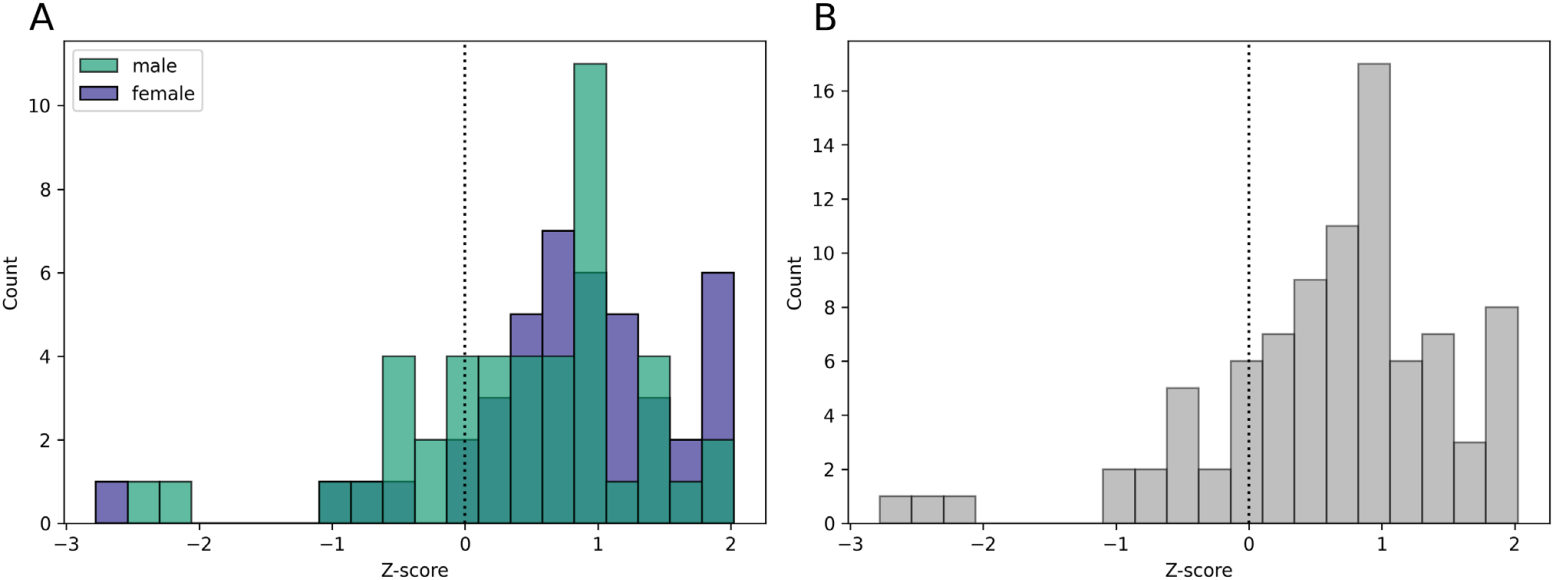
Distribution of Z-scores for timepoint 2 (post-lockdown) for the scores on the flanker task. (A) Subjects separated by sex. (B) All subjects combined. The vertical dotted line corresponds to a Z-score of zero.

**Fig. 4.**
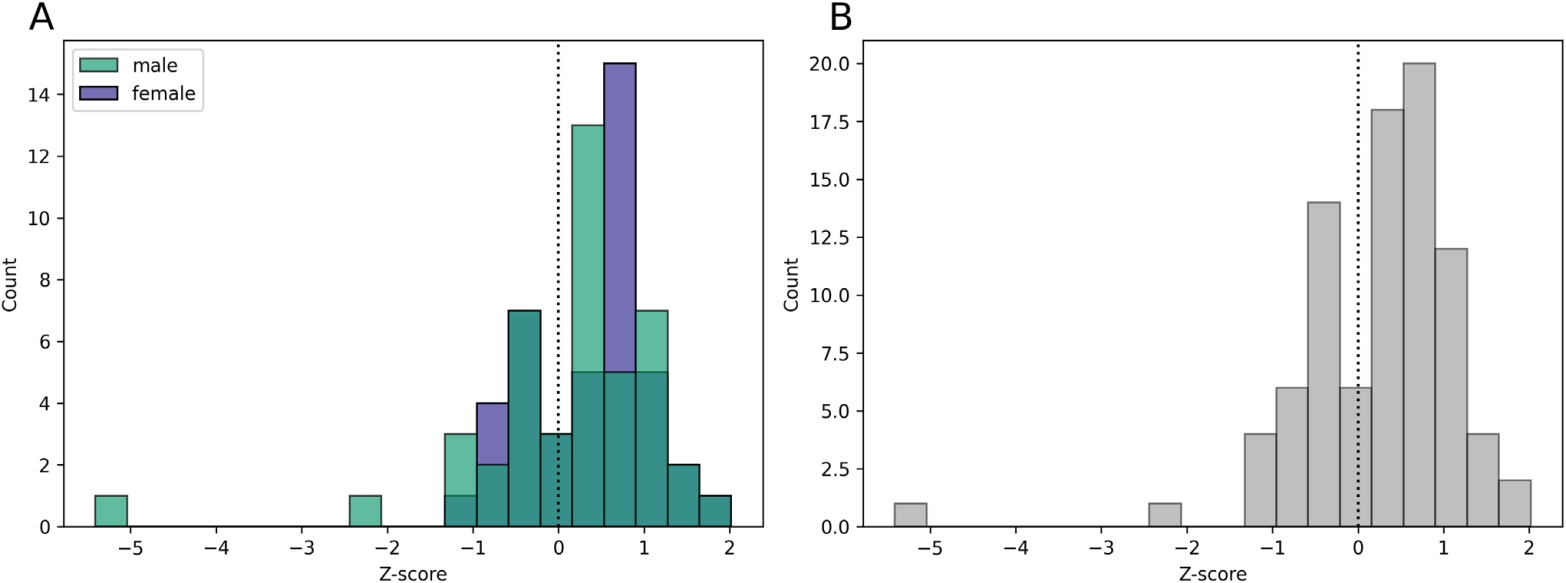
Distribution of Z-scores for timepoint 2 (post-lockdown) for the scores on the dimensional change card sort task. (A) Subjects separated by sex (B) All subjects combined. The vertical dotted line corresponds to a Z-score of zero.

Post-lockdown performance on the flanker (Fig. 3) and dimensional change card sort (DCCS; Fig. 4) tasks was significantly higher than predicted by the normative models. For the flanker test, the mean Z-score for all subjects at timepoint 2 was 0.601. The *p*-value for the one-sample *t*-test after correction for multiple comparisons was 4.24 e-8. For the DCCS, the mean Z-score for all subjects at timepoint 2 was 0.242 and the *p*-value for the one sample *t*-test after multiple comparison correction was .020. There was no effect of sex (corrected *p* = 0.22 for flanker and *p* = 0.27 for DCCS).

### 3.3 Correlations between white matter and executive function development

In the exploratory analysis of associations between brain white matter and executive function findings, no associations survived FDR correction for multiple comparisons. The strongest observed exploratory association was a negative correlation between post-lockdown flanker Z-scores and post-lockdown MD Z-scores in the callosum forceps minor (Pearson *r* = -0.297, uncorrected *p* = 0.013). Given that this finding did not survive multiple comparison correction, it should be interpreted with caution.

## 4 Discussion

We report lower MD of white matter relative to age-normative expectations, as well as higher executive function performance relative to normative expectations, in both male and female adolescents who experienced the COVID-19 pandemic. We additionally report higher FA in one tract in females and lower FA in one tract in males relative to normative expectations. In a direct comparison between the sexes, we found that in two tracts, the deviations of post-lockdown FA from normative trajectories were significantly greater in females than in males.

Lower MD and higher FA in the post-lockdown data relative to age-normative expectations are consistent with accelerated maturation of the white matter, although this interpretation requires caution as they could alternatively reflect pathological mechanisms or other processes affecting the characteristics of water diffusion. The findings support our hypotheses that white matter development in adolescents was altered by the pandemic, and that there was an effect of sex. As with our previously reported findings of accelerated gray matter maturation (Corrigan et al., 2024), the results from this study do not provide a clear explanation as to the exact factors that contributed to these findings, as the pandemic resulted in many changes, which, in addition to increased social isolation and interrupted in-person schooling, included other factors, such as alterations in sleep (Moitra & Madan, 2022), eating habits (Moitra & Madan, 2022; Pourghazi et al., 2022), and screen use (Choi et al., 2023; Li et al., 2021). In our previous study we speculated that pandemic-related acceleration in gray matter maturation may be due to stress, as there is well-established literature linking accelerated gray matter maturation to stress during early development (Colich et al., 2020). We speculate that the results from the current study may be due to stress as well. There are established mechanisms by which stress can affect white matter development (Long et al., 2021). Increases in white matter myelin or volume due to stress are consistent with the stress acceleration hypothesis (Callaghan & Tottenham, 2016), which interprets acceleration in brain maturation as an adaptation that limits the vulnerability of the developing brain to the effects of a stressful environment (Jensen et al., 2018).

Multiple previous studies have reported accelerated gray matter development in association with the COVID-19 pandemic (Corrigan et al., 2024; Gotlib et al., 2023; van Drunen et al., 2023). Gray and white matter are closely linked in both spatial location and function, and their development and maturation are interdependent (Giorgio et al., 2010; Olesen et al., 2003). For instance, in a study of gray and white matter myelination, Zika et al. (2025) reported findings suggesting that gray and white matter develop in concert during early infancy. Luo et al. (2026) have additionally reported that, in participants ranging in age from late childhood to early adulthood, changes in development of the white matter in superficial tract regions coincide with the development of the cerebral cortex. The findings of the current study, together with prior reports of accelerated gray matter maturation associated with the pandemic, are consistent with this literature supporting coordinated gray and white matter change during development.

The improvements in EF observed in the post-lockdown data were not expected, as numerous previous studies have reported declines in executive functioning associated with the COVID-19 pandemic. One reason for the difference may be the way executive functions were evaluated. Whereas the EF measures of this study were based on in-person administration of the flanker and dimensional card sort tasks, most of the previous reports were based on parent or self-reports. Another reason for the difference in findings may have to do with timing of the administration of the tests. Many of the previous studies of the effects of the COVID-19 pandemic on skills associated with executive function were administered during the pandemic, whereas this study evaluated them a year after the lockdown.

We speculate that the improvements in EF task performance observed in this study are also related to stress. Although the relationship between stress and EF is not consistent across the literature (Girotti et al., 2024; Shields et al., 2016), a number of studies of adults have reported that stress improves performance on executive function tasks such as response inhibition (Chang et al., 2020; Gao et al., 2022; Kofman et al., 2006; Schwabe et al., 2013; Spencer et al., 2024; Wu et al., 2022; Yan et al., 2024), attention (Banks et al., 2014; Qi & Gao, 2020; Qi et al., 2024), selective attention (Chajut & Algom, 2003; Shields et al., 2019), reversal learning (Wieland et al., 2023), task-switching (Kofman et al., 2006), cognitive flexibility (Delahaye et al., 2015), and working memory (Cornelisse et al., 2011; Schoofs et al., 2013; Weerda et al., 2010), including the flanker task (Qi et al., 2024; Qi & Gao, 2020; Shields et al., 2019) and the Wisconsin card sorting task (Delahaye et al., 2015). Notably, most of these studies administered a measure of acute time-limited stress; however, two of them studied the effects of a chronic stressor (Gao et al., 2022; Qi et al., 2024). Both of these studies reported improvements in flanker task performance with stress. The neurophysiological mechanisms that underlie improvements in the domain of EF under stress are not clear, but research suggests that improvements in EF subdomains may be due to improved attention (Qi & Gao, 2020; Yan et al., 2024). Both EF tasks administered in this study require inhibition, or a suppression of responses to stimuli that are not the target stimulus. Accelerated development in the ability to withhold inappropriate responses may be an advantage in the presence of environmental stressors (Schwabe et al., 2013).

We found differences in deviations for FA of white matter maturation to be greater in females as compared to males in two of the tracts we investigated. In our previous work, we also observed sex effects on gray matter development. We speculate that these sex differences result from interactions between sex and stress hormones on brain development, but this hypothesis would need to be verified with further research.

Our analysis included an exploratory evaluation of correlations between the degree of deviation of white matter development from typical trajectories with the deviations of EF scores from typical trajectories. No findings survived multiple comparison correction. The strongest association was a negative association between performance on the flanker task and MD in the callosum forceps minor. There have been previous associations of performance on the flanker task and the callosum forceps minor (Stammen et al., 2023; Tamnes et al., 2012), which connects the right and left prefrontal cortex.

Accelerated brain development during childhood and adolescence has been associated with reduced brain plasticity, which can be thought of as an adaptation to reduce the effects of an adverse environment on the developing brain. It is also associated with an increased incidence of mental health problems (Callaghan & Tottenham, 2016; Lockhart et al., 2018). The behavioral findings from this study suggest that pandemic-related alterations to the brain’s typical structural-developmental trajectory are adaptive in nature, and that this adaptation may also have beneficial aspects. The improvements in two executive function tasks suggest that the brain’s ability to adapt to the environment may be protective in nature and can enhance performance in some domains.

There are limitations to the present study. First, several sample characteristics should be considered when interpreting these findings. Compared to other multi-site studies of adolescents (Alexander et al., 2017; Jernigan et al., 2018; Somerville et al., 2018), the sample size for this study was relatively small. However, recent work examining reference cohort size in normative modeling suggests that smaller samples can yield robust models (Elleaume et al., 2026). In addition, the socioeconomic status values of our sample suggest possible over-representation of relatively advantaged participants, which may limit generalizability to more socioeconomically disadvantaged populations. Second, our data analysis was cross-sectional. Since a direct comparison of the pre- and post-lockdown measurements in the same subjects would not have allowed us to distinguish between the effects of typical aging and the effects of the pandemic, we were unable to take advantage of the longitudinal nature of the collected data. Third, we did not collect data on pandemic-related stressors, such as food insecurity or financial insecurity, which would have allowed for a better characterization of the stress experienced by the participants in this study. Fourth, the same executive function tasks were administered at two timepoints, 3 years apart, in the same study participants. Although practice effects are typically examined over shorter time intervals, their contribution to the present findings cannot be excluded. The executive function improvements observed in this study likely reflect a combination of developmental change and residual task familiarity. Fifth, we do not know whether the accelerated maturation in structural development and executive functioning observed in these subjects is permanent, or only temporary. Future work is necessary to evaluate the long-term effects of the COVID-19 pandemic on brain structure and EF skills in this population. Finally, since we did not collect medical history data at timepoint 2, we do not know whether the COVID-19 virus itself may have contributed to these findings.

In summary, the present study adds to existing evidence that the COVID-19 pandemic was linked to alterations in typical brain development in adolescents, with accelerated development being the most predominant finding. The executive function findings further suggest that these alterations may be associated with improvements in specific cognitive domains. However, because specific contributing factors were not directly measured, these deviations cannot be attributed to any single cause and likely reflect the combined influence of multiple concurrent pandemic-related changes. The pattern of accelerated white matter maturation alongside improved executive performance is consistent with stress-related adaptive processes, although alternative explanations, such as changes in sleep, schooling, or daily routines, remain plausible. Together, these findings suggest that developmental timing can shift in response to environmental conditions, potentially conferring functional advantages. Further study is needed to clarify causal pathways and determine the longer-term cognitive and mental health implications of these changes.

## Supplementary Material

Supplementary Material

## Data Availability

The data for this study and the code used in the analysis are available upon request.
