# Supplementary material for: Accelerated maturation of brain circuits and executive function in adolescents who experienced the COVID-19 pandemic
Source: Imaging Neurosci (Camb). 2026 Jul 22;4:IMAG.a.1307. doi: 10.1162/IMAG.a.1307 (PMC13392998; doi:10.1162/IMAG.a.1307)
Supplement: Supplementary Material [file IMAG.a.1307_supp.pdf]

Supplementary Materials for

Accelerated Maturation of Brain Circuits and Executive Function in Adolescents who Experienced the COVID-19 Pandemic

Neva M. Corrigan *et al.*

*Corresponding author. Email: nevao@uw.edu

Table S1. Timeline of COVID-19 pandemic restrictive measures in Washington State

Date	Measure
March 17, 2020	Washington K-12 school closures went into effect. Large public gatherings prohibited.
March 23, 2020	Washington statewide stay-at-home order issued.
December 16, 2020	Washington K-12 schools given an option for in-person learning for areas with low COVID-19 spread.
March 11, 2021	Partial openings allowed for businesses, youth sports in Washington State.
April 19, 2021	Washington K-12 schools mandated to offer at least 30% in-person instruction.
June 30, 2021	All Washington State COVID-19 restrictive measures lifted.
September 9, 2021	All Washington State K-12 schools return to in-person instruction.

Sources: King 5 News (2021); KREM News (2021); Ballotpedia (n.d.).

Performance metrics for normative models, assessed on held-out pre-COVID validation data

Normative model predictive performance for each dMRI metric and tract was assessed on held-out validation data in each of 100 repeated random train/validation splits of the pre-COVID data. Performance values for each tract were averaged across all nodes and splits. Tracts with explained variance < 0 were omitted from this table and excluded from subsequent analyses of post-lockdown data.

Table S2. Predictive Performance Metrics for Imaging Data

**Mean Diffusivity**	Explained Variance	RMSE	Pearson's R
Callosum Forceps Minor	0.083	0.029	0.324
Left Arcuate	0.035	0.025	0.292
Left Corticospinal	0.049	0.022	0.316
Left IFOF	0.075	0.027	0.317
Left ILF	0.055	0.031	0.317
Left SLF	0.070	0.028	0.318
Left Thalamic Radiation	0.084	0.023	0.351
Left Uncinate	0.093	0.029	0.343
Right Arcuate	0.020	0.024	0.297
Right Corticospinal	0.186	0.023	0.464
Right IFOF	0.099	0.027	0.373
Right SLF	0.076	0.021	0.323
Right Thalamic Radiation	0.178	0.022	0.452
Right Uncinate	0.220	0.030	0.505

**Fractional Anisotropy**	Explained Variance	RMSE	Pearson's R
Callosum Forceps Minor	0.022	0.040	0.184
Left Arcuate	0.010	0.055	0.216
Left SLF	0.017	0.059	0.225
Left Thalamic Radiation	0.009	0.046	0.195
Right Corticospinal	0.020	0.070	0.195
Right IFOF	0.021	0.053	0.161
Right SLF	0.017	0.047	0.211

Statistical Analysis of Combined-Sex Imaging Results

A supplementary analysis of post-lockdown deviations from pre-COVID norms, based on combined data from males and females, was performed. MD results are shown in Table S3. No FA findings survived multiple comparison correction.

Table S3. Mean Diffusivity (MD) findings in the post-lockdown data for combined-sex analysis

Tract	Effect size	p	FDR
Left Thalamic Radiation	-0.42	0.00	**0.00**
Right Thalamic Radiation	-0.43	0.00	**0.00**
Left Corticospinal	-0.13	0.05	0.10
Right Corticospinal	-0.17	0.04	0.08
Callosum Forceps Minor	-0.44	0.00	**0.00**
Left IFOF	-0.14	0.09	0.12
Right IFOF	-0.17	0.05	0.10
Left ILF	-0.14	0.16	0.20
Left SLF	-0.06	0.53	0.56
Right SLF	-0.02	0.77	0.77
Left Uncinate	-0.24	0.03	0.07
Right Uncinate	-0.08	0.31	0.35
Left Arcuate	-0.15	0.08	0.12
Right Arcuate	-0.13	0.09	0.12

Z-score Distributions by Sex

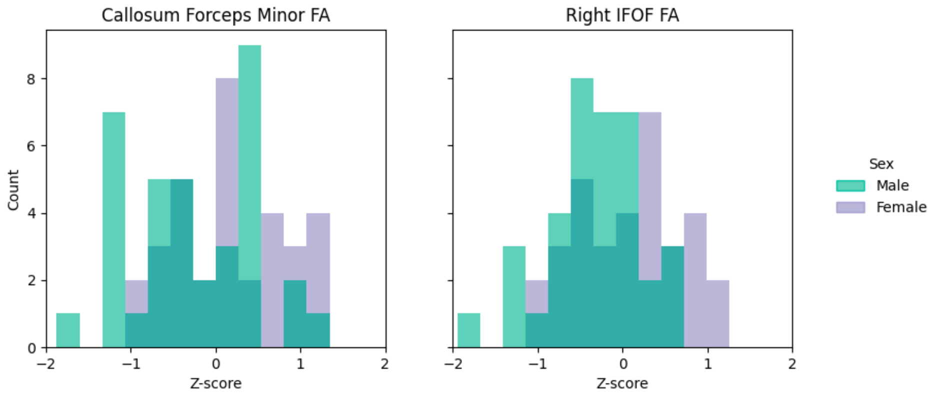

Fig. S1. FA Z-score distributions for males and females averaged across all nodes on the tracts showing statistically significant sex differences.

Executive Function Sensitivity Analysis

A sensitivity analysis was performed to evaluate the effect of using a second-order (nonlinear) spline for the normative modeling of the EF data. Results from this analysis showed that post-lockdown performance on the flanker task was significantly higher than predicted by the normative models (mean Z = 0.546, corrected p = 4.88 e-7). In contrast, post-lockdown performance on the DCCS task was not significant (mean Z = 0.117, corrected p = 0.28). There was no effect of sex (corrected p = 0.18 for flanker and corrected p = 0.20 for DCCS).

REFERENCES

King 5 News. (2021, June 30). Washington state fully reopens, ending more than a year of COVID restrictions. *King 5 News*. https://www.king5.com/article/news/local/washington-state-reopen-fully-wednesday-covid-restrictions/281-f3100a26-3121-4ed9-92d3-9272bc32b975

KREM News. (2021, June 28). COVID-19 closure timeline: Washington to reopen after nearly 15 months. *KREM News*. https://www.krem.com/article/news/health/coronavirus/timeline-of-washington-covid-19-closures/293-7b785999-81b9-467d-b004-0bececf30b1f

Ballotpedia. (n.d.). School responses in Washington to the coronavirus (COVID-19) pandemic. *Ballotpedia*. https://ballotpedia.org/School_responses_in_Washington_to_the_coronavirus_(COVID-19)_pandemic
